# Cell‐sheet delivery for wound healing through transfer‐tattoo approach: A new wound management era?

**DOI:** 10.1002/hsr2.1902

**Published:** 2024-02-15

**Authors:** Lakshmi Venkata Simhachalam Kutikuppala, Sri Harsha Boppana, Karthikeya Reddy Ramasahayam, Jaya Surya Manimekalai Krishnamurthi, Ganesh Tummala, Snehasish Mishra, Ranjan K. Mohapatra, Md. Kudrat‐E‐Zahan

**Affiliations:** ^1^ Department of General Surgery Dr YSR University of Health Sciences Vijayawada Andhra Pradesh India; ^2^ Department of Anesthesia and Critical Care Johns Hopkins School of Medicine Baltimore Maryland USA; ^3^ Department of Internal Medicine Dr YSR University of Health Sciences Vijayawada Andhra Pradesh India; ^4^ Madras Medical College Chennai Tamil Nadu India; ^5^ Department of General Medicine Dr YSR University of Health Sciences Vijayawada Andhra Pradesh India; ^6^ School of Biotechnology KIIT Deemed‐to‐be‐University Bhubaneswar Odisha India; ^7^ Department of Chemistry Government College of Engineeering Bhubaneswar Odisha India; ^8^ Department of Chemistry Rajshahi University Rajshahi Bangladesh

Dear Editor,

Assessment and management of wound healing is an unmet therapeutic challenge within the medical fraternity since it is a complex procedure where several factors in the healing process play major roles. Professor Sungjune Jung and his team created a transfer‐tattoo‐like cell‐sheet delivery in South Korea for wound management.^1^ The cell sheet that could be directly placed on the healing surface was prepared by a mechanism of interfacial cell migration. This cell engineering demonstrates multilayer stacking using various cell types, enabling the production of artificial three‐dimensional tissues with intricate multicellular patterns

Wound healing is challenging in medical sciences since its assessment and management is a complex procedure that is dependent on several intricate factors with critical roles.^2^ A major problem in wound management is the likely presence of colonizing microbes on the wound, although not in all wounds. The local necrotic or devitalized tissue is removed during wound debridement and the microbial load is reduced, a crucial phase in wound healing. Debridement includes autolytic, enzymatic, biological, surgical/sharp, and mechanical techniques. Hydrosurgery, ultrasound therapy, and plasma‐mediated bipolar radiofrequency ablation therapy are recent advancements that may provide an effective alternative to traditional wound management in critical situations. Although these approaches are costly, they could be viewed as more efficient interventions than conventional techniques, recommended especially for chronic wounds.^3^ A South Korean study team led by Professor Sungjune Jung created a cell‐sheet delivery similar to transfer tattoo to manage wounds.^4^ The interfacial cell migration allows the cell sheet that was put directly on the “wounded” skin surface to set. The cell suspension injection is usually used to expedite an injured tissue to regenerate. Yet, this technique frequently fails to guarantee the best possible result of adherence of the injected cells to the region, resulting in insufficient regeneration benefits.^4^, ^5^

Cell‐sheet delivery mechanism seems unique for improved cellular adhesion responsiveness. However, transferring the typical cell sheet system to the target cells and separating the cell sheets from the surface is done by unsafe external induced stimuli. Films with multiple layers are softer and thicker and have less adhesive strength. This issue could be resolved by raising the pH at which polyelectrolyte films were made, and stiffer films with improved adhesive characteristics can also be generated. Cell types such as hepatocytes, fibroblasts, and endothelial cells have been grown and separated as cell sheets from the surface.^6^

## 1 CELL‐SHEET DELIVERY MECHANISM

Research on cytoskeletal dynamics and pharmacological aspects during the cell‐transfer process revealed that interfacial cell migration occurred by using the pre‐existing cell transmembrane surface proteins to connect to a targeted surface. For a stable cell attachment during culture and effective cell transfer to the target surface, it was suggested that the UV‐treated parylene appropriately provided intermediate degree cell adhesiveness. Parylene is chemically inert, crystalline, transparent, and thermoplastic profoundly used as a coating material in biomedicines. It is considered hemocompatible and perhaps is also a beneficial substance for in vitro cell culture studies.^7^ The adaptability of this cell‐transfer procedure in an array of cell (such as HDFn, MRC‐5, MC3T3‐E1, A549, C2C12, MDCK‐II, and HULEC‐5a) types was demonstrated.^1^, ^5^ Interfacial cell migration with differing adhesion preferences between the surfaces served as the foundation for the revolutionary cell‐sheet engineering. Establishing and reversing cell polarity after it is transferred, involving the distinct ability of 3D multilayer stacking, freeform design, and application to curved surfaces, are also critical.^5^

## 2 THERAPEUTIC POTENTIAL OF CELL‐SHEET DELIVERY

External stimulation and detachment procedures by cell‐sheet engineering developed by Park et al. seemed an unnecessary procedure as the cells migrate naturally.^4^ This revolutionary technology possesses great biocompatibility, process simplicity, and workflow convenience levels as compared to the earlier methods.^1^ Research revealed for the first time that vertical migration of cells across two varying surfaces is also possible beyond their known horizontal migration. This vertical migration technique that formed the basis of their platform for cell delivery was termed “interfacial cell migration.” Cell‐sheet delivery engineering also demonstrated the multilayer stacking capacity, enabling the production of 3D tissues and the option to use an array of cell types, permitting the fabrication of artificial tissues with intricate multicellular patterns.^2^, ^8^

Application of cell‐sheet delivery technology to the cutaneous and subcutaneous wound healing and skin‐tissue regeneration illustrates its therapeutic potential for the benefit of hospitalized patients with deformed skin in particular and the medical sciences (especially, reconstructive and cosmetic surgery) in general. Further research to validate its potential and fool‐proof application in various clinical conditions is recommended.

## AUTHOR CONTRIBUTIONS

**Lakshmi Venkata Simhachalam Kutikuppala**: Conceptualization; writing—original draft. **Sri Harsha Boppana**: Conceptualization; writing—original draft. **Karthikeya Reddy Ramasahayam**: Writing—original draft. **Jaya Surya Manimekalai Krishnamurthi**: Writing—original draft. **Ganesh Tummala**: Writing—original draft. **Snehasish Mishra**: Writing—original draft. **Ranjan K. Mohapatra**: Writing—review and editing; supervision. **Md. Kudrat‐E‐Zahan**: Writing—original draft.

## CONFLICT OF INTEREST STATEMENT

The authors declare no conflict of interest.

## Data Availability

Data sharing is not applicable to this article as no new data were created or analyzed in this study.
